# Increased adipose tissue heme levels and exportation are associated with altered systemic glucose metabolism

**DOI:** 10.1038/s41598-017-05597-2

**Published:** 2017-07-13

**Authors:** José María Moreno-Navarrete, Amaia Rodríguez, Francisco Ortega, Sara Becerril, Mònica Sabater-Masdeu, Jessica Latorre, Wifredo Ricart, Gema Frühbeck, José Manuel Fernández-Real

**Affiliations:** 1 Department of Diabetes, Endocrinology and Nutrition, Institut d’Investigació Biomèdica de Girona (IdIBGi), Hospital of Girona ‘Dr JosepTrueta’, Carretera de França s/n, 17007 Girona, Spain; 2 Centro de Investigación Biomédica en Red de Fisiopatología de la Obesidad y Nutrición (CIBEROBN), Instituto de Salud Carlos III (ISCIII), Madrid, Spain; 3 Metabolic Research Laboratory, Clínica Universidad de Navarra, Pamplona, 31008 Spain; 4 Department of Medicine, Universitat de Girona, Girona, 17007 Spain

## Abstract

Iron status is known to be associated with the physiology of adipose tissue (AT). We aimed to investigate AT heme and expression of heme exporter (FLVCR1) in association with obesity and type 2 diabetes (T2D). Substantial amounts of *FLVCR1* mRNA and protein levels were detected in AT, being significantly increased in subjects with T2D, and positively correlated with fasting glucose, fasting triglycerides and with circulating markers of iron stores (serum ferritin, blood hemoglobin and hematocrit). In both visceral (VAT) and subcutaneous AT (SAT), increased heme levels were found in subjects with T2D. Reinforcing these associations, *FLVCR1* mRNA levels were positively linked to fasting glucose in an independent cohort. Longitudianlly, the percent change of *FLVCR1* positively correlated with the percent change in fasting glucose (r = 0.52, p = 0.03) after bariatric surgery-induced weight loss. High-fat diet-induced weight gain in rats did not result in significant changes in AT *Flvcr1* mRNA but, remarkably, the expression of this gene positively correlated with fasting glucose and negatively with insulin sensitivity (QUICKI). Altogether, these findings showed a direct association between *FLVCR1* mRNA levels and hyperglycemia, suggesting that increased adipose tissue heme exportation might disrupt, or is the consequence of, impaired systemic glucose metabolism during the progression to T2D.

## Introduction

Heme constitutes a relevant form of functional iron in the human body, as well as two-thirds of the average person’s iron intake in developed countries^1^. Epidemiological studies have substantiated increased risks of type-2 diabetes associated with high heme intake^2–6^. Even though non-heme iron is the predominant form of dietary iron, heme iron is more easily absorbed^2^. In fact, the intake of heme iron, but not of non-heme iron, has been positively associated with body iron stores^2, 6^, and increased body iron stores have been extensively demonstrated in type 2 diabetes^7, 8^. Mechanistically, excess of intracellular heme levels are degraded by heme oxygenase resulting in increased free iron^9^. In fact, increased heme oxygenase activity was found in patients with type 2 diabetes in association with body iron stores^9^. Furthermore, intracellular free heme excess catalyzes the formation of reactive oxygen species (ROS), promoting oxidative stress^10, 11^. Increased tissue iron deposition and iron-induced oxidative stress might result in increased beta-cells apoptosis, hepatic dysfunction and insulin resistance, and in consequence, to promote the progression of type 2 diabetes^6^.

Feline Leukemia Virus subgroup C Receptor 1 (FLVCR1) is a plasma membrane heme exporter that ensures the maintenance of appropriate intracellular heme concentration^12–14^. The depletion of FLVCR1 led to excess heme content in erythroid progenitors, and in consequence, increased cytoplasmic ROS and apoptosis, whereas restoring *FLVCR1* gene expression returned to normal erythropoiesis, demonstrating that excess of intracellular heme levels disrupted this cellular process^12–14^. Recent studies demonstrated the importance of FLVCR1 in the maintenance of heme homeostasis in other tissues, including intestine^15^ and liver^16^. Interestingly, the disruption of FLVCR1 in these tissues resulted in increased tissue heme accumulation in parallel to increased heme oxygenase 1 (HMOX1) and markers of oxidative stress and iron accumulations (ferroportin and ferritin levels)^15, 16^.

Increased iron^17–19^, markers of iron accumulation^17, 20–22^ and HMOX1 mRNA and protein levels^23–25^ have been demonstrated in mice and human adipose tissue in association with adipose tissue dysfunction (increased inflammation and decreased adipogenesis). *In vitro* experiments in mouse cells revealed increasing heme biosynthesis during adipocyte differentiation^26^. Increased intracellular heme accumulation resulted in oxidative stress and adipocyte hypertrophy^27^. In fact, heme-induced HMOX1 activity decreased adipocyte differentiation of human preadipocytes, and attenuated glucose uptake, mitochondrial function in parallel to increased inflammation and markers of oxidative stress and iron accumulation in human adipocytes^25^.

To the best of our knowledge, FLVCR1 and heme levels have not been previously explored in human adipose tissue. Considering these intriguing studies^17–27^ and the importance of FLVCR1 in the maintenance of tissue heme homeostasis^12–16^, we aimed to investigate cross-sectionally (in two cohorts) the expression of *FLVCR1* according to obesity and type 2 diabetes (T2D), and longitudinally the effects of weight gain (in rats) and weight loss (bariatric surgery of morbidly obese subjects) on AT *FLVCR1* mRNA levels. Adipose tissue heme levels were also examined.

## Results

### Heme levels and heme exporter-related gene expression in human AT

**Figure 1:**
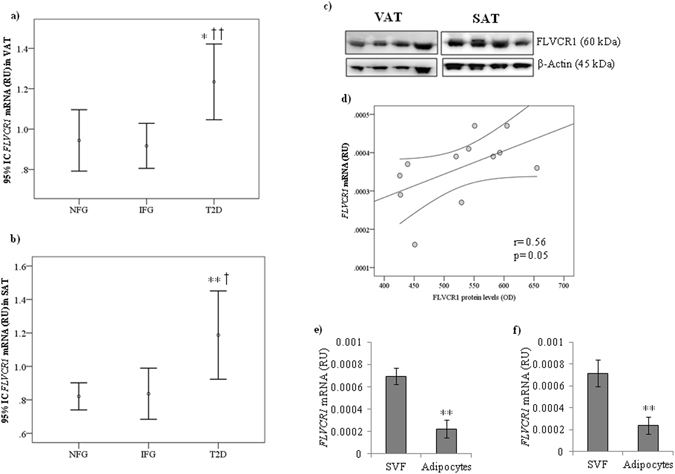
*FLVCR1* gene expression in VAT **(a)** and SAT **(b**) according to fasting glucose status and type 2 diabetes in cohort 1 [NFG (n = 80), IFG (n = 29) and T2D (n = 19)]. *p < 0.05 and **p < 0.01 compared with NFG participants. ^†^p < 0.05 and ^††^p < 0.01 compared with IFG participants. RU, relative units of gene expression. Gene expression was calculated by 2^−∆∆Ct^ method. (**c**) Representative immunoblot of FLVCR1 protein levels in VAT (n = 7) and SAT (n = 5) from NFG participants. (**d**) Bivariate correlation between *FLVCR1* gene expression and FLVCR1 protein levels (normalized by β-actin) (n = 12). (**e**,**f**) *FLVCR1* gene expression in adipose tissue fractions (SVF vs adipocytes) in VAT (**e**) and SAT (f) (n = 5). **p < 0.01 compared with SVF.

Anthropometrical and clinical parameters of the study subjects are shown in Table 1. Substantial amounts of heme exporter (*FLVCR1*) mRNA and protein levels were detected in both VAT and SAT (Fig. 1a–c). *FLVCR1* mRNA was positively correlated with FLVCR1 protein levels (r = 0.56, p = 0.05; Fig. 1d). Adipose tissue fraction analysis indicated increased *FLVCR1* mRNA levels in stroma vascular fraction (SVF) in comparison to adipocytes (Fig. 1e,f) in both VAT and SAT.

**Table 1:** Anthropometric and clinical parameters of study subjects from cohort 1 and cohort 2.

*Cohort 1*	NFG	IFG	T2D	p (ANOVA)
SAT				
n	80	29	19	
Sex (men/women)	19/61	8/21	5/14	
Age (years)	45.2 ± 12.2	48.5 ± 11.3	48.3 ± 9.9	0.3
BMI (kg/m^2^)	37.7 ± 10.7	43.3 ± 7.9*	42.5 ± 6.6*	0.01
Fasting glucose (mg/dL)	88.4 ± 8.3	112.1 ± 10.1*	189.1 ± 48.1**^†^	<0.0001
HDL-cholesterol (mg/dL)	56.9 ± 15.5	54.1 ± 15.1	47.8 ± 11.6	0.1
Fasting triglycerides (mg/dL)	90 (73–130)	121 (101.5–157.7)*	147 (114–200)*	0.001
Serum ferritin (ng/mL)	39 (9–69.4)	70 (30–125)	102.5 (32.9–188.6)	0.2
VAT				
n	79	27	17	
Sex (men/women)	21/58	6/21	5/12	
Age (years)	46.1 ± 12.5	48.5 ± 10.7	50.7 ± 10.6	0.3
BMI (kg/m^2^)	37.3 ± 10.9	43.1 ± 8.8*	42.9 ± 6.1*	0.01
Fasting glucose (mg/dL)	89.3 ± 8.1	113.2 ± 10.1*	182.4 ± 46.1**^†^	<0.0001
HDL-cholesterol (mg/dL)	55.9 ± 15.8	52.8 ± 14.8	48.8 ± 10.4	0.2
Fasting triglycerides (mg/dL)	93 (75–139)	134 (100.5–180)*	147 (174–116)*	0.002
Serum ferritin (ng/mL)	44.8 (8.9–87.1)	71.5 (44.3–157.5)	88 (46–176.2)	0.2
*Cohort 2* n	50			
Sex (men/women)	9/41			
Age (years)	47.7 ± 8.7			
BMI (kg/m^2^)	44.9 ± 6.6			
Fasting glucose (mg/dL)	96 (89.7–108.2)			
HDL-cholesterol (mg/dL)	47.6 ± 12.1			
Fasting triglycerides (mg/dL)	115 (82–156.2)			
Serum ferritin (ng/mL)	55 (22.5–124)			

NFG, normal fasting glucose; IFG, impaired fasting glucose; T2D, type 2 diabetes; BMI, body mass index. Bold values indicate statistically significant p values.

*p < 0.05 and **p < 0.01 compared with NFG participants, performing Bonferroni post hoc test.

^†^p < 0.05 compared with IFG participants, performing Bonferroni post hoc test.

In both VAT (Fig. 1a) and SAT (Fig. 1b), *FLVCR1* gene expression was significantly increased in patients with T2D when compared with AT form patients with impaired fasting glucose or with normal fasting glucose levels, and expression of this gene was positively correlated with fasting glucose (Table 2). Expression of the *FLVCR1* gene was associated with other T2D-associated metabolic traits, including positive correlations with fasting triglycerides in both SAT and VAT, and circulating markers of iron stores (serum ferritin, blood hemoglobin and hematocrit) only in VAT (Table 2). Negative correlations with HDL-cholesterol in SAT and expression of adipogenesis (*ADIPOQ*) and insulin signaling-related genes (*SLC2A4, IRS1*) (Table 2) in VAT were also observed.

**Table 2:** Bivariate correlations among VAT and SAT *FLVCR1* gene expression, clinical parameters and expression of adipose tissue- related genes in cohort 1 and cohort 2.

	Cohort 1				Cohort 2			
	VAT (N = 123)		SAT (N = 128)		VAT (N = 50)		SAT (N = 50)	
	r	p	r	p	r	p	r	p
Age (years)	0.048	0.5	0.05	0.6	0.02	0.8	−0.10	0.4
BMI (kg/m^2^)	0.093	0.3	-0.14	0.11	0.17	0.2	0.06	0.6
Fasting glucose (mg/dl)	0.28	**0.006**	0.26	**0.003**	0.31	**0.02**	0.27	**0.04**
Total-cholesterol (mg/dl)	0.16	0.1	−0.07	0.4	−0.06	0.7	−0.11	0.4
LDL-cholesterol (mg/dl)	0.14	0.2	−0.07	0.4	−0.16	0.2	−0.10	0.4
HDL-cholesterol (mg/dl)	−0.10	0.3	−0.32	**<0.0001**	0.10	0.5	−0.15	0.2
Fasting triglycerides (mg/dl)	0.22	**0.03**	0.25	**0.008**	0.17	0.2	0.11	0.4
Serum ferritin (ng/ml)	0.29	**0.006**	0.20	0.06	−0.11	0.5	−0.04	0.7
Blood hemoglobin (g/dl)	0.26	**0.008**	0.16	0.1	0.22	0.1	−0.04	0.7
Hematocrit (%)	0.22	**0.03**	0.12	0.2	0.27	**0.04**	0.01	0.9
*ADIPOQ* (R.U.)	−0.28	**0.01**	0.11	0.4	0.19	0.2	−0.01	0.9
*SLC2A4* (R.U.)	−0.31	**0.005**	−0.07	0.5	−0.05	0.7	−0.20	0.1
*IRS1* (R.U.)	−0.25	**0.02**	0.15	0.1	−0.24	0.06	−0.06	0.6
*TNF* (R.U.)	0.06	0.5	0.08	0.4	−0.13	0.4	0.04	0.7
*IL6* (R.U.)	0.09	0.4	0.18	0.1	0.17	0.3	−0.11	0.4
*ITGAX* (R.U.)	0.06	0.5	0.08	0.4	0.12	0.5	0.06	0.7
*FOXP3* (R.U.)	0.10	0.3	−0.08	0.4	0.11	0.5	−0.12	0.5
Hemin in AT (µg/mg) (N = 41)	0.34	**0.04**	0.37	**0.03**	—	—	—	—

VAT, visceral adipose tissue; SAT, subcutaneous adipose tissue; R.U., relative units of gene expression. Bold values indicate statistically significant P values.

**Figure 2:**
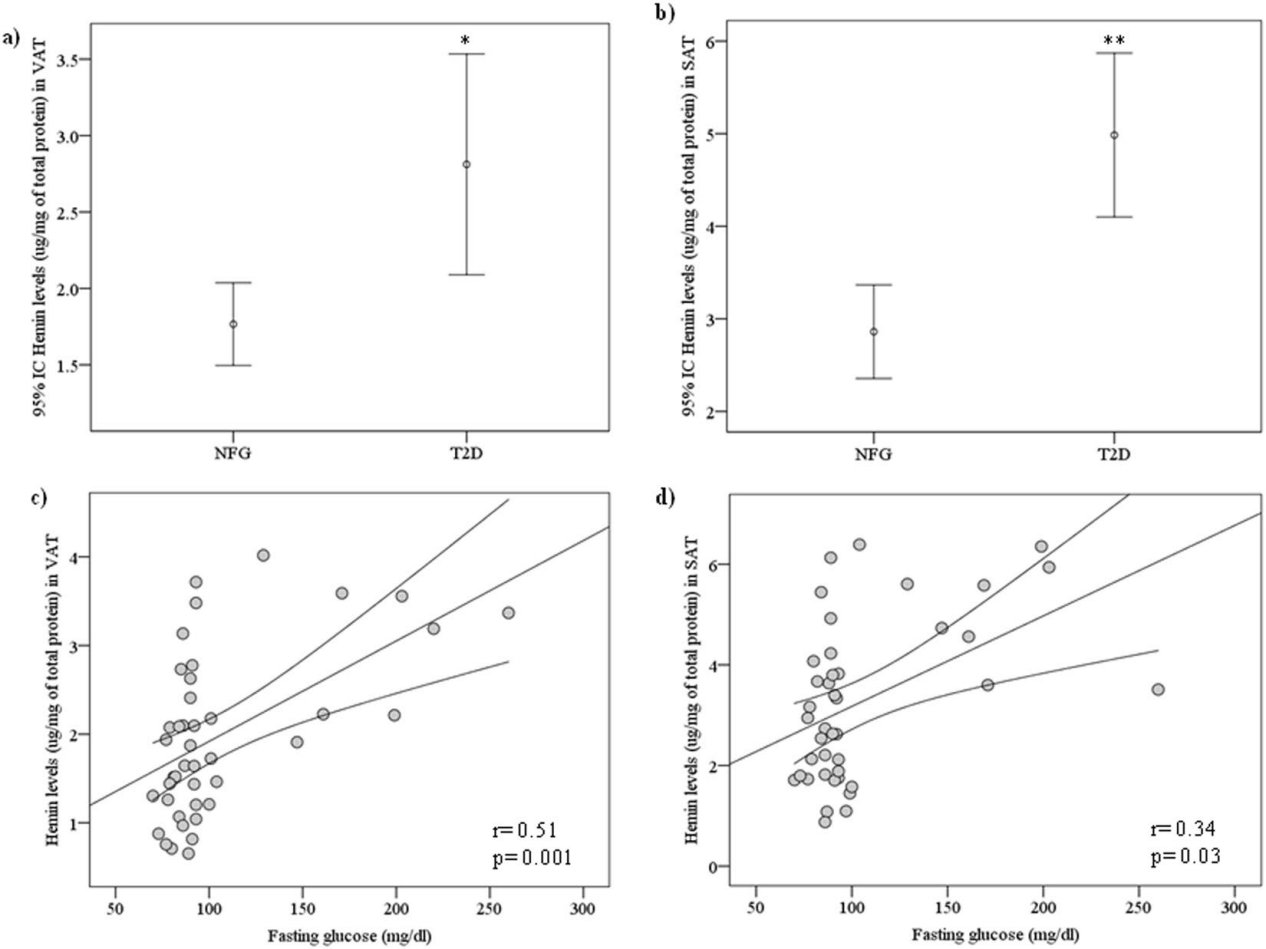
Hemin levels in VAT (**a**) and SAT **(b)** according to type 2 diabetes in cohort 1 [NFG (n = 32) and T2D (n = 9)]. *p < 0.05 and **p < 0.01 compared with NFG participants. (**c,d**) Bivariate correlation between adipose tissue hemin levels and fasting glucose (n = 41) in both VAT **(c**) and SAT (**d)**.

To investigate a possible association between adipose tissue FLVCR1 and heme levels, adipose tissue heme levels were measured in a subgroup of 41 [32 NFG and 9 T2D (Table 3)] participants from cohort 1. In both SAT and VAT, adipose tissue heme levels were detected at substantial amounts and were significantly increased in patients with T2D (Fig. 2a,b). Heme levels positively correlated with fasting glucose (r = 0.51, p = 0.001 in VAT; r = 0.34, p = 0.03 in SAT, Fig. 2c,d). VAT and SAT heme levels were positively correlated with *FLVCR1* mRNA levels (r = 0.34, p = 0.04 in VAT; r = 0.37, p = 0.03 in SAT, Table 2).

**Table 3:** Anthropometric and clinical parameters of study subjects from subcohort 1.

	NFG	T2D	p (ANOVA)
n	32	9	
Sex (men/women)	8/24	1/8	
Age (years)	44.1 ± 10.1	51 ± 5.9	0.06
BMI (kg/m^2^)	42.7 ± 8.2	46.8 ± 3.2	0.1
Fasting glucose (mg/dL)	85.1 ± 7.1	184 ± 40.2	<0.0001
HDL-cholesterol (mg/dL)	53.4 ± 15.3	50.6 ± 10.5	0.6
Fasting triglycerides (mg/dL)	87 (75.5–128)	118 (100–229.5)	0.1
Serum ferritin (ng/mL)	28.9 (8.4–63)	70 (41.1–110)	0.02

Since heme oxygenase 1 (*HMOX1)* gene expression is known to be strongly induced by heme levels [23–25], *HMOX1* mRNA levels were also analyzed in this subgroup from cohort 1 (Table 3). Adipose tissue heme levels positively correlated with *HMOX1* gene expression (r = 0.32, p = 0.04 in VAT; r = 0.36, p = 0.02 in SAT, Suppl Fig. 1A,B).

### Replication in an independent cohort

Anthropometrical and clinical parameters of these subjects are shown in Table 1. Reinforcing the above described associations, *FLVCR1* gene expression was positively correlated with fasting glucose (r = 0.31, p = 0.02 in VAT; r = 0.27, p = 0.04 in SAT) (Table 2).

### Effects of bariatric surgery-induced weight loss

**Figure 3:**
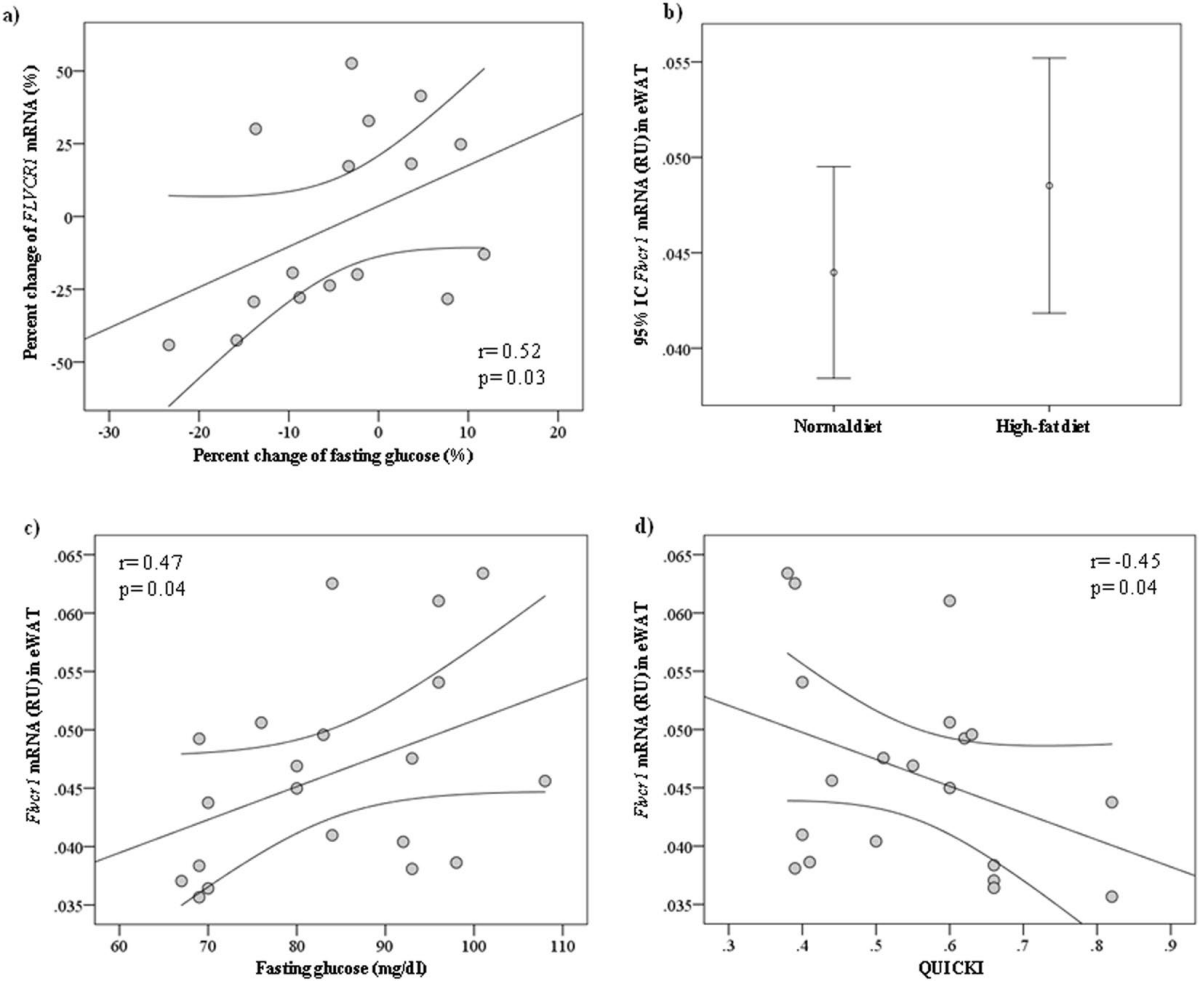
(**a**) Bivariate correlation between the change of *FLVCR1* gene expression and the change of fasting glucose 2 years after bariatric surgery (Cohort 3, n = 16)). (**b**) The effect of high-fat diet (HFD) on eWAT *Flvcr1* gene expression in rat experiments [normal diet (n = 10) and high fat diet (n = 10)]. (**c,d**) Bivariate correlation between eWAT *Flvcr1* gene expression and fasting glucose **(c**) or QUICKI (**d**) (n = 20).

Bariatric surgery-induced weight loss did not result in significant changes in *FLVCR1* gene expression (Suppl Table 1). However, the percent change of *FLVCR1* gene expression was positively correlated with the percent change of fasting glucose (r = 0.52, p = 0.03, Fig. 3a). Fasting glucose decreased (95.2 ± 7.1 *vs* 86.3 ± 5.2, p = 0.002) in 11 participants and increased (88.8 ± 10.1 *vs* 95.4 ± 12.1, p = 0.01) in 5 subjects two years after surgery.

### Effects of weight gain in rats

In rats, comparing those fed with HFD *vs* normal diet (Suppl Fig. 1C–E), no significant differences on *Flvcr1* gene expression in epididymal white adipose tissue (eWAT) were observed (Fig. 3b). Nonetheless, expression of *Flvcr1* gene was positively correlated with fasting glucose (r = 0.47, p = 0.04; Fig. 3c) and negatively with QUICKI (r = −0.45, p = 0.04; Fig. 3d). *Flvcr1* was not significantly correlated with *Il6* (r = 0.29, p = 0.2) and *Slc2a4* (r = −0.21, p = 0.3) mRNA levels in eWAT.

## Discussion

This study demonstrated substantial amounts of heme and *FLVCR1* mRNA and protein levels in both VAT and SAT. The main finding of the present study was that adipose tissue heme and *FLVCR1* mRNA levels were increased in AT from patients with type 2 diabetes in direct proportion to fasting glucose levels, but not in association with BMI. Taking into account the well-demonstrated relationship between high diet heme iron uptake and increased risk of type 2 diabetes^2–6^, this study suggests a relationship between adipose tissue export of heme iron and type 2 diabetes. In line with these results, the change of *FLVCR1* mRNA levels ran in parallel with the change of fasting glucose independently of weight loss. Fasting glucose decreased in some participants and increased in others 2 years after surgery. These discrepancies could be explained by the fact that no patient with type 2 diabetes was included in the current study. It is known that the effects of bariatric surgery on fasting glucose reduction are more pronounced in patients with type 2 diabetes^28^. After weight gain, *Flvcr1* mRNA levels were positively associated with fasting glucose and negatively with insulin sensitivity (QUICKI) in rats, but not with fatness. In cohort 1, both SAT and VAT *FLVCR1* gene expression was also associated with fasting triglycerides, and with serum ferritin and decreased mRNA levels of adipogenic markers in VAT. In line with this, increased susceptibility to iron-induced insulin resistance and dysfunctional adipogenesis has been demonstrated in VAT^20, 29^. Increased intracellular heme accumulation is known to result in dysfunctional and hypertrophic adipocytes^27^, suggesting that adipocyte heme excess might contribute to the link between adipocyte hypertrophy and type 2 diabetes^30–33^. In addition *FLVCR1* mRNA was negatively correlated with expression of insulin signaling-related genes (*SLC2A4* and *IRS1*). Decreased *SLC2A4* and *IRS1* gene expression in adipose tissue are associated with insulin resistance^34^.

In adipose tissue fraction, *FLVCR1* mRNA levels were increased in SVF vs adipocytes. Considering that FLVCR1 is required for T cell development and survival^35^, highly expressed in macrophages^13, 36^, and decreased under inflammatory conditions^36^, the relationship between adipose tissue FLVCR1 and markers of macrophages [*ITGAX* ^37^], regulatory T lymphocytes [*FOXP3* ^38^] or inflammation (*TNF*, *IL6*) was explored. Of note, adipose tissue *FLVCR1* were not associated with *ITGAX*, *FOXP3*, *TNF* or *IL6* mRNA levels, suggesting that adipose tissue *FLVCR1* mRNA was not attributed to inflammatory cells.

Adipose tissue heme levels were confirmed using *HMOX1* mRNA levels, another surrogate marker of intracellular heme levels^39^. By different ways, HMOX1 (heme degradation)^23–25^ and FLVCR1 (heme exportation)^13–16^ might impact on intracellular heme levels. Increased HMOX1 gene and protein expression has been recently demonstrate in obese participants, but not in association with type 2 diabetes^24, 25, 40, 41^, whereas FLVCR1 was increased specifically in patients with type 2 diabetes (current findings).

Heme biosynthesis is required for adipocyte differentiation^26^. Increased fat accretion results in raised heme levels in adipose tissue, enhanced heme-induced HMOX1 expression and activity, free iron accumulation^25^, proinflammatory activity and, in consequence, insulin resistance and hyperglycemia. In bone marrow, depletion of FLVCR1 (i.e. lack of heme exportation), with the attendant heme accumulation, leads to cell damage and apoptosis^42^. Efficient heme exportation is also crucial in maintaining intestinal homeostasis^15^. The situation regarding adipose tissue, reported for the first time in the present manuscript, seems to be different from these two cellular models. The current study has shown how impaired glucose metabolism was associated with increased heme levels in adipose tissue in parallel to raised expression of *FLVCR1* and *HMOX1*. These data suggest that heme exportation and degradation in adipose tissue is possibly induced in diabetogenic conditions to counteract intracellular heme levels, preventing their negative effects on free iron-induced oxidative stress and cellular damage^10, 11^. However, further functional experiments are necessary to clarify the relevance of FLVCR1 in adipocyte physiology

In conclusion, intracellular heme excess in human adipocytes might result in enhanced export of heme iron, and in alterations in systemic glucose metabolism. Possibly, iron accumulation in other tissues [liver^19^, muscle^43^ and brain^44^] would also worsen hyperglycemia and type 2 diabetes. The current study is focused on adipose tissue, but considering the importance of other insulin-dependent tissues (including liver and skeletal muscle) on glucose metabolism, additional studies exploring the relationship between heme exportation and glucose metabolism in liver and skeletal muscle are required. Further molecular and cellular studies should be designed to decipher the mechanism underlying the relationship between heme exportation and glucose metabolism.

## Methods

### Human adipose tissue samples

Adipose tissue samples were obtained from two independent cohorts. In cohort 1 (N = 251), visceral (VAT, N = 123) and subcutaneous (SAT, N = 128) adipose tissue samples from participants with normal body weight and different degrees of obesity, body mass index (BMI) ranging from 20 to 68 kg/m^2^, recruited at the Endocrinology Service of the Hospital Universitari Dr. Josep Trueta (Girona, Spain) were analyzed. In a second cohort of non-diabetic morbidly obese (cohort 2, BMI > 35 kg/m^2^) subjects, 50 paired SAT and VAT samples were studied. All subjects were of Caucasian origin and reported that their body weight had been stable for at least three months before the study. Liver and renal diseases were specifically excluded by biochemical work-up. All subjects gave written informed consent, validated and approved by the ethical committee of the Hospital Universitari Dr. Josep Trueta after the purpose of the study was explained to them. All methods were performed in accordance with the relevant guidelines and regulations. Adipose tissue samples were obtained from SAT and VAT depots during elective surgical procedures (cholecystectomy, surgery of abdominal hernia and gastric bypass surgery). Adipose tissue samples were washed, fragmented and immediately flash-frozen in liquid nitrogen before being stored at −80 °C.

The isolation of adipocyte and stromal vascular fraction cells (SVF) was performed from 6 SAT (n = 5) and VAT (n = 5) non-frozen adipose tissue samples as described previously^22^. These samples were washed three to four times with phosphate-buffered saline (PBS) and suspended in an equal volume of PBS supplemented with 1% penicillin-streptomycin and 0.1% collagenase type I warmed to 37 °C. The tissue was placed in a shaking water bath at 37 °C with continuous agitation for 60 minutes and centrifuged for 5 minutes at 300 to 500 *g* at room temperature. The supernatant, containing mature adipocytes, was recollected. The pellet was identified as the SVF. Isolated mature adipocytes and SVF were stored at −80 °C for gene expression analysis.

### Study of the effects of fat mass reduction induced by bariatric surgery

Sixteen subjects who underwent bariatric surgery via Roux-en-Y gastric bypass (RYGB) at the Hospital Universitari Dr. Josep Trueta (Girona, Spain) were part of an ongoing study^21, 25^. Inclusion criteria were age between 30 and 60 years, BMI ≥ 35 kg/m^2^ and ability to understand the study protocol. Exclusion criteria were: (i) baseline T2D; (ii) use of medications able to interfere with insulin action; (iii) acute infection process 4 weeks prior to sample collection; (iv) history of a chronic systemic disease or other serious chronic associated illness. Adipose tissue samples from the SAT depot were obtained during bariatric surgery. Postoperative samples of SAT were obtained by subcutaneous biopsy at the mesogastric level 2 years after surgery. Fasting blood samples were obtained at the same day of the biopsy. All subjects gave written informed consent, validated and approved by the ethical committee of the Hospital Universitari Dr. Josep Trueta, after the purpose of the study was explained to them. All methods were performed in accordance with the relevant guidelines and regulations.

### Heme levels measurement

In a subgroup of 41 [32 with normal fasting glucose (NFG) and 9 with T2D)] participants from cohort 1, adipose tissue heme levels were measured using the hemin assay kit (ab65332, Abcam plc, Cambridge, UK) according to the manufacturer’s instructions, with a coefficient of variation <10%.

### Analytical methods

Serum glucose concentrations were measured in duplicate by the glucose oxidase method using a Beckman glucose analyser II (Beckman Instruments, Brea, California). Intraassay and interassay coefficients of variation were less than 4% for all these tests. HDL-cholesterol was quantified by an homogeneous enzymatic colorimetric assay through the cholesterol esterase/cholesterol oxidase/peroxidase reaction (Cobas HDLC3). Total serum triglycerides were measured by an enzymatic, colorimetric method with glycerol phosphate oxidase and peroxidase (Cobas TRIGL). We used a Roche Hitachi Cobas c 711 instrument to perform the determinations. Serum ferritin was determined by Microparticle Enzyme ImmunoAssay (AXSYMTM; Abbot Laboratories, Abbott Park, IL), with a coefficient of variation intra- and interassay <6%.

### Effects of weight gain in rats

Four-week-old male Wistar rats (n = 20) (breeding house of the University of Navarra) were housed in a room with controlled temperature (22 ± 2 °C), relative humidity (50 ± 10%), ventilation (at least 15 complete changes of air/h), and 12:12 light-dark cycle (lights on at 8:00 am). Rats were housed in individual cages and were fed *ad libitum* during an average of 6 months either a normal chow diet [ND (12.1 kJ/g: 4% fat, 48% carbohydrate and 14% protein; diet 2014, Harlan, Teklad Global Diets, Harlan Laboratories Inc., Barcelona, Spain); n = 10] for comparative purposes or a high-fat diet [HFD (23.0 kJ/g: 60% fat, 27% carbohydrate and 14% protein; diet F3282, Bio-Serv, Frenchtown, NJ, USA); n = 10] to induce obesity. Body weight and food intake were recorded on a regular basis to monitor progression of diet-induced obesity. After an overnight fast, rats were sacrificed by decapitation and the epididymal white adipose tissue (eWAT) depots were carefully dissected out, weighed, frozen in liquid nitrogen, and stored at –80 °C. All experimental procedures conformed to the European Guidelines for the Care and Use of Laboratory Animals (directive 2010/63/EU) and were approved by the Ethical Committee for Animal Experimentation of the University of Navarra (049/10). All methods were performed in accordance with the relevant guidelines and regulations.

### RNA expression

RNA purification was performed using RNeasy Lipid Tissue Mini Kit (QIAgen, Izasa SA, Barcelona, Spain) and the integrity was checked by Agilent Bioanalyzer (Agilent Technologies, Palo Alto, CA). Gene expression was assessed by real time PCR using an LightCycler^®^ 480 Real-Time PCR System (Roche Diagnostics SL, Barcelona, Spain), using TaqMan^®^ technology suitable for relative genetic expression quantification. The RT-PCR reaction was performed in a final volume of 12 μL. The cycle program consisted of an initial denaturing of 10 min at 95 °C then 40 cycles of 15 sec denaturizing phase at 95 °C and 1 min annealing and extension phase at 60 °C. A threshold cycle (Ct value) was obtained for each amplification curve and a ∆Ct value was first calculated by subtracting the Ct value for human cyclophilin A (*PPIA*) RNA from the Ct value for each sample. Fold changes compared with the endogenous control were then determined by calculating 2^−∆Ct^, so gene expression results are expressed as expression ratio relative to PPIA gene expression according to manufacturers’ guidelines. Primer/probe sets used were: feline leukemia virus subgroup C cellular receptor 1 (*FLVCR1*, Hs01067777_m1); tumor necrosis factor (*TNF*, Hs00174128_m1); adiponectin (*ADIPOQ*, Hs00605917_m1); solute carrier family 2 (facilitated glucose transporter), member 4 (*SLC2A4* or *GLUT4*, Hs00168966_m1); Insulin receptor substrate 1 (*IRS1*, Hs00178563_m1); integrin subunit alpha X (*ITGAX* or *CD11C*, Hs00174217_m1); forkhead box P3 (*FOXP3*, Hs01085834_m1); Interleukin 6 (*IL6*, Hs00985639_m1); heme oxygenase 1 (*HMOX1*, Hs01110250_m1); and Peptidylprolyl isomerase A (cyclophilin A) (4333763, *PPIA* as endogenous control). In rats, primer/probe sets used were feline leukemia virus subgroup C cellular receptor 1**, (** *Flvcr1*, Rn01411620_m1), interleukin 6 (*Il6*, Rn01410330_m1), solute carrier family 2 (facilitated glucose transporter), member 4 (*Slc2a4* or *Glut4*, Rn00562597_m1) and Eukaryotic 18S rRNA (4331182) as endogenous control.

### Protein preparation and western blot

To explore whether FLVCR1 protein is detected in human adipose tissue in proportion to *FLVCR1* mRNA levels, FLVCR1 protein was analysed in 12 samples according to the amount of available adipose tissue. Adipose tissue proteins were extracted directly in radio immnunoprecipitation assay (RIPA) buffer (0.1% SDS, 0.5% sodium deoxycholate, 1% Nonidet P-40, 150 mmol/L NaCl, and 50 mmol/L Tris-HCl, pH 8.00), supplemented with protease inhibitors (1 mmol/L phenylmethylsulfonyl fluoride). Cellular debris and lipids were eliminated by centrifugation of the solubilized samples at 14000 *g* for 10 min at 4 °C, recovering the soluble fraction. Protein concentration was determined using the RC/DC Protein Assay (Bio-Rad Laboratories, Hercules, CA). RIPA protein extracts (25 μg) were separated by SDS-PAGE and transferred to nitrocellulose membranes by conventional procedures. Membranes were immunoblotted with anti-FLVCR antibody (ab70838, Abcam plc, Cambridge, UK) and β-actin antibodies (sc-47778, Santa Cruz Biotechnology, CA, USA). Anti-rabbit IgG and anti-mouse IgG coupled to horseradish peroxidase was used as a secondary antibody. Horseradish peroxidase activity was detected by chemiluminescence, and quantification of protein expression was performed using Scion Image software.

### Statistical analyses

Statistical analyses were performed using the SPSS 12.0 software. Unless otherwise stated, descriptive results of continuous variables are expressed as mean and SD for Gaussian variables or median and interquartile range for non-Gaussian variables. Parameters that did not fulfill normal distribution criteria were log transformed to improve symmetry for subsequent analyses. The relation between variables was analyzed by simple correlation (using Spearman’s and Pearson’s tests). ANOVA and unpaired Student’s *t*-tests were used to compare clinical variables, heme levels and *FLVCR1* gene expression relative to obesity and T2D.

### Availability of materials and data

All data generated or analysed during this study are included in this published article (and its Supplementary Information files).

## Electronic supplementary material

Supplementary Information
