# Supplementary material for: Increased adipose tissue heme levels and exportation are associated with altered systemic glucose metabolism
Source: Sci Rep. 2017 Jul 13;7:5305. doi: 10.1038/s41598-017-05597-2 (PMC5509649; doi:10.1038/s41598-017-05597-2)
Supplement: Supplementary file 1 — Supplementary Information [file 41598_2017_5597_MOESM1_ESM.pdf]

Increased adipose tissue heme levels and exportation are associated with altered systemic glucose metabolism

José María Moreno-Navarrete, Amaia Rodríguez, Francisco Ortega, Sara Becerril, Mònica Sabater-Masdeu, Jessica Latorre, Wifredo Ricart, Gema Frühbeck, José Manuel Fernández- Real

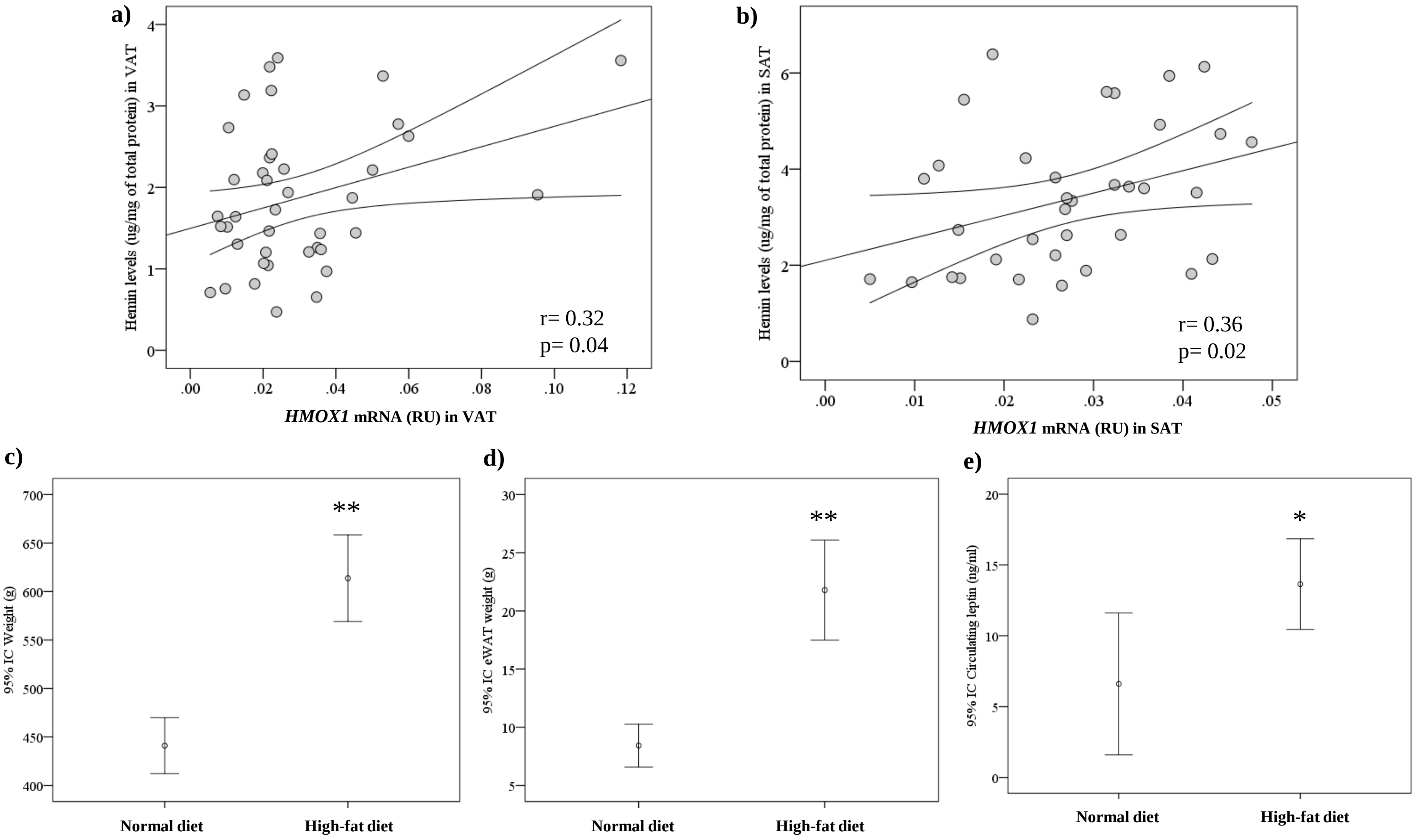

Supplemental figure 1. a-b) Bivariate correlation between adipose tissue hemin levels and *HMOX1* gene expression in both VAT **(a)** and SAT **(b)** in subcohort1. **c-e)** Effect of high-fat diet (HFD) on weight **(c)**, eWAT weight **(d)** and circulating leptin **(e)** in rat experiments. *p<0.05 and **p<0.01 compared with normal diet.
